# Maternal creatine homeostasis is altered during gestation in the spiny mouse: is this a metabolic adaptation to pregnancy?

**DOI:** 10.1186/s12884-015-0524-1

**Published:** 2015-04-14

**Authors:** Stacey J Ellery, Domenic A LaRosa, Michelle M Kett, Paul A Della Gatta, Rod J Snow, David W Walker, Hayley Dickinson

**Affiliations:** 1 The Ritchie Centre, MIMR-PHI Institute of Medical Research, 27-31 Wright St, Clayton, Melbourne, 3168 Australia; 2 Department of Obstetrics & Gynaecology, Monash University, Monash Medical Centre, Clayton, Melbourne, Australia; 3 Department of Physiology, Monash University, Clayton Campus, Melbourne, Australia; 4 Centre for Physical Activity and Nutrition, Deakin University, Burwood Campus, Melbourne, Australia

**Keywords:** Nutrition, Cellular energy, Metabolism, Pregnancy, Fetal development, Spiny mouse

## Abstract

**Background:**

Pregnancy induces adaptations in maternal metabolism to meet the increased need for nutrients by the placenta and fetus. Creatine is an important intracellular metabolite obtained from the diet and also synthesised endogenously. Experimental evidence suggests that the fetus relies on a maternal supply of creatine for much of gestation. However, the impact of pregnancy on maternal creatine homeostasis is unclear. We hypothesise that alteration of maternal creatine homeostasis occurs during pregnancy to ensure adequate levels of this essential substrate are available for maternal tissues, the placenta and fetus. This study aimed to describe maternal creatine homeostasis from mid to late gestation in the precocial spiny mouse.

**Methods:**

Plasma creatine concentration and urinary excretion were measured from mid to late gestation in pregnant (n = 8) and age-matched virgin female spiny mice (n = 6). At term, body composition and organ weights were assessed and tissue total creatine content determined. mRNA expression of the creatine synthesising enzymes arginine:glycine amidinotransferase (AGAT) and guanidinoacetate methyltransferase (GAMT), and the creatine transporter (CrT1) were assessed by RT-qPCR. Protein expression of AGAT and GAMT was also assessed by western blot analysis.

**Results:**

Plasma creatine and renal creatine excretion decreased significantly from mid to late gestation (P < 0.001, P < 0.05, respectively). Pregnancy resulted in increased lean tissue (P < 0.01), kidney (P < 0.01), liver (P < 0.01) and heart (P < 0.05) mass at term. CrT1 expression was increased in the heart (P < 0.05) and skeletal muscle (P < 0.05) at term compared to non-pregnant tissues, and creatine content of the heart (P < 0.05) and kidney (P < 0.001) were also increased at this time. CrT1 mRNA expression was down-regulated in the liver (<0.01) and brain (<0.01) of pregnant spiny mice at term. Renal AGAT mRNA (P < 0.01) and protein (P < 0.05) expression were both significantly up-regulated at term, with decreased expression of AGAT mRNA (<0.01) and GAMT protein (<0.05) observed in the term pregnant heart. Brain AGAT (<0.01) and GAMT (<0.001) mRNA expression were also decreased at term.

**Conclusion:**

Change of maternal creatine status (increased creatine synthesis and reduced creatine excretion) may be a necessary adjustment of maternal physiology to pregnancy to meet the metabolic demands of maternal tissues, the placenta and developing fetus.

## Background

Maternal metabolic adaptations are required during pregnancy to meet the nutrient demands of the growing placenta and fetus [1]. These changes include shifts into anabolic metabolism during the first and second trimesters [2]. This allows for fat accumulation in the mother, to be used as an energy source for maternal tissues during the third trimester, when a switch to catabolic metabolism takes place to ensure an enhanced nutrient supply to the growing fetus [2-6]. These adaptations to meet fetal requirements for growth encompass large changes in maternal glucose, carbohydrate, amino acid, lipid and fatty acid metabolism [2,7]. An inability of the maternal system to make these adjustments and ensure appropriate nutrient supply for both maternal and fetal tissues may underpin some of the obstetric complications associated with poor fetal growth and placental insufficiency [8].

While creatine is thought to be an essential metabolite for the growing fetus [9,10], whether maternal creatine metabolism is altered in pregnancy has received little attention. Creatine and its intracellular phosphorylated derivative, phosphocreatine, have an important role in cellular ATP turnover [11]. Creatine is readily obtained from a diet of animal products and is also synthesised by the body through the production of guanidinoacetate (GAA), catalysed by the enzyme arginine:glycine aminotransferase (AGAT) predominately in the kidney, followed by the methylation of GAA, mainly in the liver, to produce creatine [12]. Once synthesised or absorbed creatine is moved into tissues by the creatine transporter-1 protein (CrT1) [13] where ~75% is phosphorylated and used as a source of phosphate for the regeneration of ATP [14].

Creatine has been shown to cross the human placenta [10]. However the source of this creatine is not known. In rats, AGAT, GAMT, and CrT1 are expressed in most tissues of the embryo from early in gestation [15]. Yet studies in the spiny mouse, a precocial species that has a number of reproductive similarities to human pregnancy in terms of *in utero* organ development [16-18] and endocrine function [19], identified that creatine was accrued during gestation by the placenta and fetal tissues, but that the fetus had limited capacity for creatine synthesis until 0.9 of gestation [20]. This suggests that creatine supply to the placenta and fetus in mammals that give birth to well-developed young is maternal in origin for much of gestation. This raises questions about how the maternal system provides for the proposed increase in need for creatine during pregnancy. Thus, the objective of this study was to describe maternal creatine homeostasis during gestation in the spiny mouse.

## Methods

### Spiny mice

All experiments were approved in advance by Monash University Animal Ethics Committee and conducted in accordance with the Australian Code of Practice for the Care and Use of Animals for Scientific Purposes. Spiny mice (*Acomys cahirinus*) were bred and housed as previously described [21]. Pregnant and virgin female spiny mice were used to conduct this study. Control females and near-term pregnant dams (37 days gestation [dGA]; term is 39 days) were killed by cervical dislocation and the brain, liver, kidney, heart and gastrocnemius muscle were excised, weighed, snap frozen and stored at −80°C.

### Body composition

After post mortem whole frozen bodies (non-pregnant n = 6; pregnant n = 6 (excluding the uterus and fetal tissues for the pregnant cohort)) were subjected to Dual-energy X-ray absorptiometry (DEXA; Lunar PIXImus, GE Medical Systems, Wisconsin), for assessment of lean tissue mass, bone mineral density (BMD) and tissue fat mass [22].

### Urine collection

Non-pregnant females (n = 6), and pregnant dams (n = 6) at 23dGA and 35dGA were placed in a metabolic cage apparatus (Monash Scientific Glass, Dandenong, Victoria) for 12 hours (h) during the dark cycle (19:00–07:00) to facilitate the collection of urine for measurement of creatine. Food and water intake were also measured during this time.

### Plasma collection

Non-pregnant female spiny mice (n = 6) and pregnant dams (23dGA, 35dGA and term) (n = 6-9/time-point) were anesthetised and whole blood was collected by cardiac puncture, before the spiny mice were killed by cervical dislocation. Whole blood was centrifuged at 4°C and 3000 *g* for 10 mins. Plasma was then frozen and stored at −20°C.

### Measuring creatine

Tissues (brain, liver, kidney, heart, gastrocnemius muscle; 3–4 mg) were freeze-dried, powdered and extracted with 0.5 mol/L perchloric acid and 1 mmol/L ethylenediaminetetraacetic acid (EDTA) before being neutralized with 2.1 mol/L potassium hydrogen carbonate. Plasma and urine were extracted with 1.5 M perchloric acid (50 μl acid/100 μl sample) before being neutralized with 2.1 mol/L potassium hydrogen carbonate. Extracted samples were used to determine total creatine content by enzymatic analysis of creatine and phosphocreatine with fluorometric detection as previously described [9,23,24]. Wet:dry tissue weight ratios were used to calculate estimated total creatine content per organ for kidney, liver, brain and heart samples. The calculated urinary excretion rate of creatine was based on the volume of urine collected over the 12 h period.

### Gene expression

Real-time quantitative polymerase chain reaction (RT-qPCR) was used to determine the mRNA expression of the creatine transporter-1 (CrT1) *(SLC6A8)* [GenBank: NM_ 133987.2] and creatine synthesising enzymes AGAT *(GATM)* [GenBank: NM_025961] and *GAMT* [GenBank: NM_010255.3] in the brain, liver, kidney, heart and gastrocnemius muscle as described previously [20]. Cyclophilin A *(peptidylprolyl isomeraseA; PPIA)* [GenBank: NM_017101.1] was used as the housekeeping gene. Primer sequences are shown in Table 1. Total RNA was extracted using RNeasy Kits (Qiagen, Glen Forrest, Australia) and reverse transcribed, according to manufacturer’s instructions (Promega, Madison, Wisconsin). RT-qPCR was completed using a Stratagene MX3000p thermal cycler system with SYBR green PCR Mastermix (Applied Biosystems, Carlsband, California). Cycling conditions were: 95°C 10mins; 95°C 15secs, 60°C 1 min ×40 cycles; 95°C 5secs, 60°C 15secs, 95°C 15secs ×40 cycles. Data obtained from qPCR were analysed using the ^ΔΔ^CT method [20,25], with results expressed relative to non-pregnant controls.

**Table 1:** **Primer Sequences**

Gene	Accession number (GenBank)	Forward prime sequence (5′-3′)	Reverse primer sequence (3′-5′)	Source
**CrT1** ***(SLC6A8)***	NM_133987.2	TCCTGGCACTCATCAACAG	ATGAAGCCCTCCACACCTAC	[20]
**AGAT** ***(GATM)***	NM_025961	TCACGCTTCTTTGAGTACCG	TCAGTCGTCACGAACTTTCC	[20]
**GAMT**	NM_010255.3	TGGCACACTCACCAGTTCA	AAGGCATAGTAGCGGCAGTC	[20]
**Cyclophilin A** ***(PPIA)***	NM_017101.1	CTGATGGCGAGCCCTTG	CTGCTGTCTTTGGAACTTTGTC	[20]

Oligonucleotide primer sequences used for the amplification and RT-qPCR analysis of creatine synthesizing enzymes and the creatine transporter.

### Protein expression

Frozen tissues (~10 mg) were solubilised in 12.5% Tris–HCl (pH 6.8), 10% Glycerol, 20% of 0.1 SDS/ dH_2_O and 10% protease inhibitor in dH_2_O overnight. Samples were centrifuged and protein concentration of supernatants determined by the bicinchoninic acid assay, following manufacturers instructions (BCA Protein Assay Kit#23225, Thermo Scientific, Scoresby, Australia). Aliquots of samples at a final protein concentration of 40 μg were separated by SDS-PAGE using Novex NuPAGE precast Tris-base 4-12% gels according to manufacturer’s instructions (Life Technologies, Mulgrave, Victoria). To account for inter-gel variability, samples from all groups were included on each gel and protein samples extracted from an adult spiny mouse kidney (AGAT) and liver (GAMT) used as an internal control across all gels. Protein was transferred to PVDF membranes, blocked for 1-h in 5% skim milk buffer (SMB) and incubated overnight in 1:70 anti-AGAT (Biorbyt, GATM antibody, orb28345, Cambridgeshire, United Kingdom, band size 47 kDa) or 1:250 anti-GAMT in 2.5% SMB (an affinity purified mouse monoclonal antibody, made through injection of the antigenic peptide N-terminal aa 125–145, band size 26 kDa; Monash Antibody Technologies Facility, Melbourne, Australia). Membranes were then incubated for 1-h with fluorescent secondary antibodies (Anti-Mouse IgG(H + L) Daylight™ 680 Conjugate or Anti-Rabbit IgG(H + L) Daylight™ 800 Conjugate; Cell Signalling Technologies®, Danvers, USA), before being scanned using the LiCOR® OdysseyCLx® Imaging System (Millennium Science, Mulgrave, Victoria). Membranes were then re-probed as above for GAPDH (G8795, Sigma-Aldrich, Castle Hill, NSW, band size 37 kDa), which was used as the loading control. Protein expression was determined by densitometry using Image Studio Lite™ software. Results are presented relative to non-pregnant controls. *Note:* due to the lack of a specific CrT1 antibody we were unable to perform western blots for this protein [26,27].

### Data analysis and statistics

Data are presented as means ± SEM. A Students t-Test for two independent samples was used to compare body composition, organ weights, tissue creatine content, and expression of creatine synthesising enzymes between pregnant and non-pregnant animals. A One-Way ANOVA with Bonferroni’s correction for multiple comparisons was used to assess plasma creatine concentration and urinary creatine excretion, by comparing the difference at two time-points during pregnancy to the non-pregnant controls. Prism 6 Graphpad software™ was used to perform statistical comparisons.

## Results

### Body composition and organ weights

At term the bone mineral density of pregnant spiny mice was significantly lower (14%) compared to non-pregnant females (Table 2; P < 0.05). Maternal lean tissue mass was increased with pregnancy by 16% (Table 2; P < 0.01), with no evident change in tissue fat mass (Table 2). Kidney mass was 26% greater at term in the pregnant spiny mouse compared to non-pregnant controls (Table 3; P < 0.01). Liver mass of pregnant spiny mice was also greater than controls by 29% (Table 3; P < 0.01) and hypertrophy of the heart in pregnant spiny mice was evident with a 12% increase in mass (Table 3; P < 0.05). No change in brain weight was observed between non-pregnant and pregnant spiny mice (Table 3).

**Table 2:** **Whole Body Dual X-ray Absorptiometry**

Dual X-ray Absorptiometry	Non-pregnant	Pregnant	Statistics
**BMD (g/cm** ^**2**^ **)**	0.066 ± 0.001	0.057 ± 0.001	**<0.05**
**Tissue- Lean Mass (g)**	24.9 ± 0.6	29.6 ± 1.3	**<0.01**
**Tissue- Fat Mass (g)**	8.7 ± 0.6	9.0 ± 0.6	**NS**

The effect of pregnancy on bone mineral denisty (BMD; g/cm^2^), tissue lean mass (g) and tissue fat mass (g). Values are means ± SEM; n = 6-8/group. Statistical analysis Students t-Test. Significance P ≤ 0.05.

**Table 3:** **Organ Weights**

Organs	Non-pregnant	Pregnant	Statistics
**Kidney (mg)**	225.5 ± 11.0	305.1 ± 8.7	**<0.01**
**Liver (g)**	0.899 ± 0.001	1.26 ± 0.05	**<0.01**
**Heart (mg)**	119.5 ± 9.1	134.7 ± 4.1	**<0.05**
**Brain (mg)**	823.8 ± 12.9	864.6 ± 10.7	**NS**

The weight (mg or g) of non-pregnant and term pregnant spiny mouse kidney, liver**,** heart and brain. Values are means ± SEM; n = 6-8/group for organ weights. Statistical analysis Students t-Test. Significance P ≤ 0.05.

### Plasma and urinary creatine concentration

**Figure 1:**
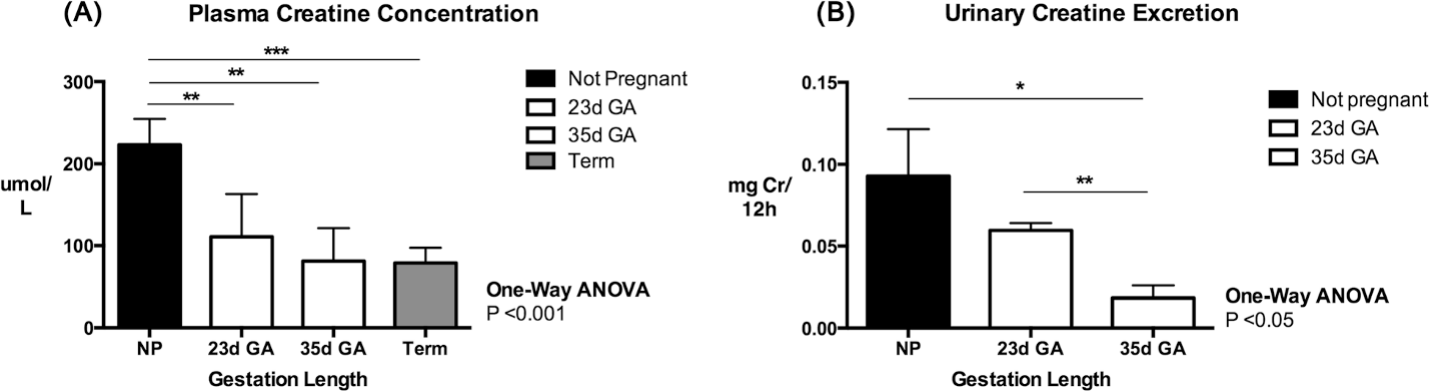
Maternal Plasma Creatine Concentration and Urinary Creatine Concentration from Mid to Late Gestation. **(A)** Plasma creatine concentration (μmol/L) of non-pregnant controls and pregnant spiny mice at 23d GA, 35d GA and term. **(B)** Urinary creatine excretion (mgCr/12 h) in non-pregnant and pregnant spiny mice at 23d GA and 35d GA. Values are means ± SEM; n = 6-8/group. Statistical analysis One-Way ANOVA with Bonferroni Multiple Comparisons. Significance P ≤ 0.05; *P <0.05, **P < 0.01, ***P < 0.001.

Plasma creatine levels were significantly lower in pregnant compared to non-pregnant spiny mice (Figure 1A; P < 0.001) with the greater part of this decrease having occurred by 23dGA (0.58 gestation). Urinary creatine excretion was significantly lower in pregnant spiny mice than non-pregnant controls (Figure 1B; P < 0.05), with creatine excretion decreasing significantly between 23dGA and 35dGA (P < 0.01). No difference in food intake, from which dietary creatine consumption was calculated, was noted between non-pregnant and pregnant spiny mice at 23dGA or 35dGA (data not shown).

### AGAT and GAMT expression

**Figure 2:**
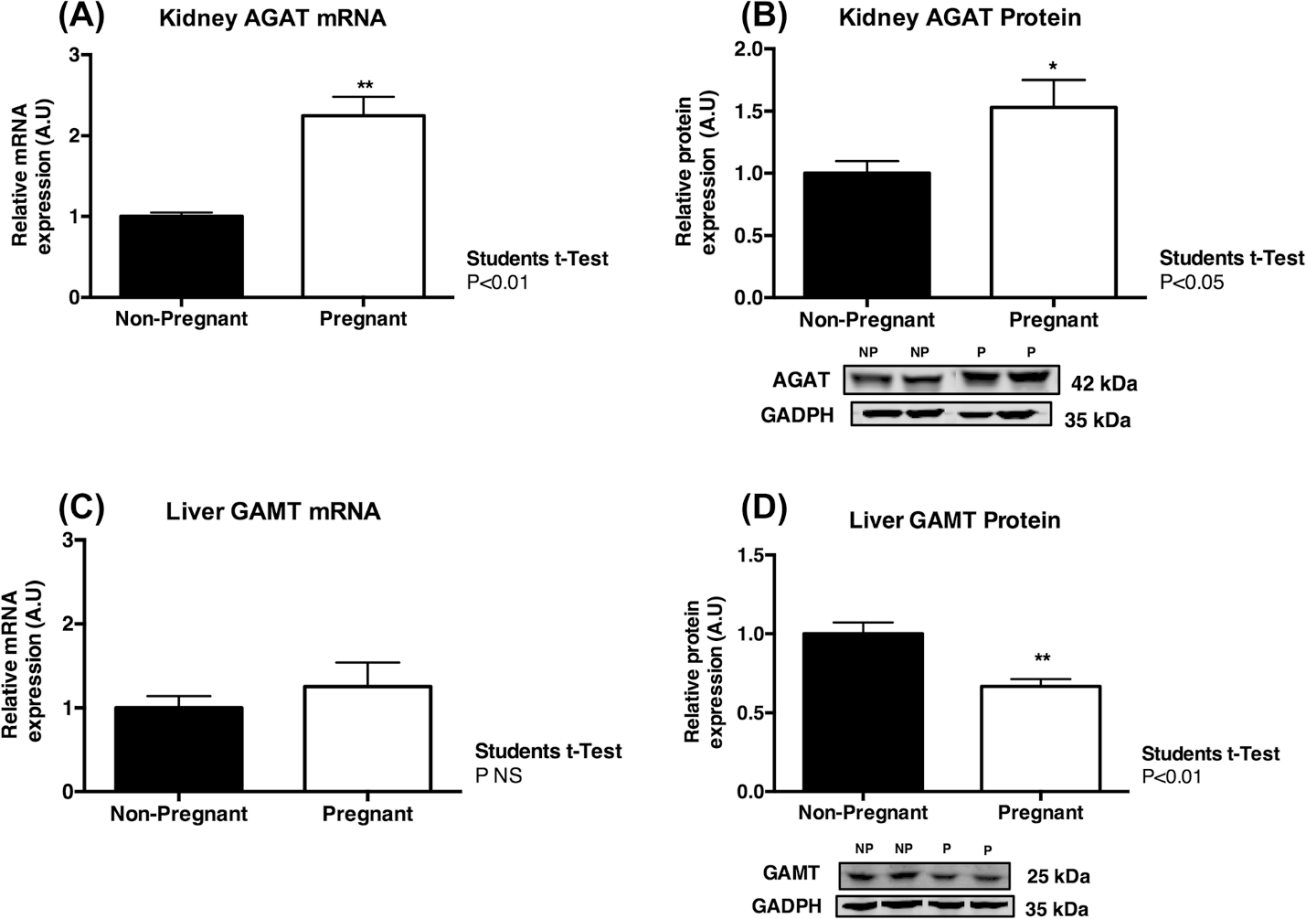
Maternal AGAT and GAMT Expression in the Kidney and Liver. Maternal kidney AGAT mRNA **(A)** and protein **(B)** expression significantly increased at term, compared to non-pregnant controls. No change in liver GAMT mRNA **(C)** was observed, however GAMT protein was decreased at term **(D)**. Data are expressed relative to the house keeping gene/protein. Values are means ± SEM; n = 6-8/group. Statistical analysis Students t-Test. Significance P < 0.05; *P <0.05.

Renal expression of AGAT, measured as mRNA and protein, were both significantly increased during pregnancy (Figure 2A & B; P < 0.01, P < 0.05, respectively). In contrast to the kidney, AGAT mRNA expression in the heart was lower in pregnant dams compared to non-pregnant controls (Table 4; P < 0.01), and there was a strong trend for heart AGAT protein expression to be lower with pregnancy (P = 0.054). AGAT mRNA expression was also significantly lower in the brain (P < 0.01) and gastrocnemius muscle (P < 0.01) of term pregnant dams compared to their non-pregnant counterparts, but with no significant change in the amount of AGAT protein present in these tissues (Table 4).

**Table 4:** **Tissue AGAT and GAMT Expression**

	Brain			Heart			Gastrocnemius muscle		
	Non-pregnant	Pregnant	Stats	Non-pregnant	Pregnant	Stats	Non-pregnant	Pregnant	Stats
**AGAT mRNA**	1.00 ± 0.06	0.48 ± 0.10	**<0.01**	1.00 ± 0.25	0.20 ± 0.02	**<0.01**	1.00 ± 0.11	0.40 ± 0.03	**<0.01**
**AGAT Protein**	1.00 ± 0.12	0.73 ± 0.18	**NS**	1.00 ± 0.26	0.46 ± 0.07	**0.054**	1.00 ± 0.45	1.23 ± 0.65	**NS**
**GAMT mRNA**	1.00 ± 0.15	0.23 ± 0.05	**<0.001**	1.00 ± 0.29	0.68 ± 0.05	**NS**	1.00 ± 0.13	0.78 ± 0.10	**NS**
**GAMT Protein**	1.00 ± 0.46	0.20 ± 0.07	**0.05**	1.00 ± 0.37	2.27 ± 0.35	**<0.05**	1.00 ± 0.42	0.77 ± 0.28	**NS**

mRNA and protein expression of AGAT and GAMT in the brain, heart and skeletal muscle of non-pregnant and pregnant spiny mice at term. Data are expressed relative to the house keeping gene/protein. Values are means ± SEM; n = 6/group. Statistical analysis Students t-Test. Significance P ≤ 0.05.

Analysis of hepatic GAMT identified no difference in mRNA expression between the pregnant and non-pregnant groups (Figure 2C), however GAMT protein expression was significantly decreased in the liver of term pregnant spiny mice compared to non-pregnant controls (Figure 2D; P < 0.01). GAMT mRNA (P < 0.001) and protein (P < 0.05) were both lower in the maternal brain at term compared to the brain in non-pregnant spiny mice (Table 4). Pregnancy was also associated with an increase in GAMT protein expression in maternal cardiac homogenates at term (Table 4; P < 0.01).

### Expression of CrT1 and tissue creatine content

**Figure 3:**
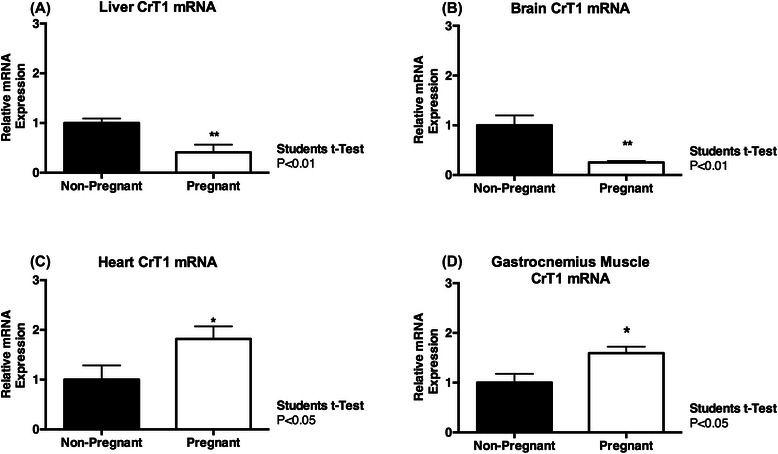
Maternal Creatine Transporter Expression. Creatine transporter 1 (CrT1) mRNA expression was reduced in the liver **(A)** and brain **(B)** of pregnant spiny mice at term. An increase in CrT1 mRNA expression, relative to non-pregnant controls, was observed in the heart **(C)** and gastrocnemius muscle **(D)** of term pregnant spiny mice. Data are expressed relative to the house keeping gene/protein. Values are means ± SEM; n = 6-8/group. Statistical analysis Students t-Test. Significance P ≤ 0.05; *P <0.01.

Cardiac CrT1 mRNA expression was significantly higher in pregnant dams compared to non-pregnant controls (Figure 3C; P < 0.05), as was the creatine content of the heart (Table 5; P < 0.05). While pregnancy had no effect on creatine content in the liver (Table 5), hepatic CrT1 mRNA was significantly lower in pregnant dams compared to non-pregnant controls (Figure 3A; P < 0.001). The creatine content in the brain was also not different between pregnant and non-pregnant females (Table 5), although brain CrT1 mRNA expression was significantly decreased in pregnant dams (Figure 3B; P < 0.01). Up-regulation of CrT1 mRNA in the gastrocnemius muscle was also observed with pregnancy (Figure 3D; P < 0.05), but creatine content in the gastrocnemius muscle was not different between pregnant and non-pregnant spiny mice (Table 5). The renal expression of CrT1 mRNA was not different between pregnant and non-pregnant spiny mice, however a significant increase in kidney creatine content was detected with pregnancy (Table 5; P < 0.001).

**Table 5:** **Tissue Creatine Content**

Tissue creatine content	Non-pregnant	Pregnant	Statistics
	***Estimated Total Creatine per Organ (nmol)***		
**Kidney**	520.9 ± 48.36	912.61 ± 48.36	<0.001
**Liver**	2432.4 ± 548.3	2554.1 ± 557.8	NS
**Brain**	14443.0 ± 399.9	12933.7 ± 1109.5	NS
**Heart**	1540.4 ± 107.7	1899.9 ± 72.36	<0.05
	***Total Creatine (mmol/kg Dry Weight)***		
**Gastrocnemius Muscle**	136.1 ± 9.4	124.1 ± 3.0	NS

Estimated total creatine content per organ (nmol). Data calculated from total creatine content, wet/dry weight ratio and wet mass per organ, except for the gastrocnemius muscle (mmol/kg dry mass). Values are means ± SEM; n = 6/group. Statistical analysis Students t-Test. Significance P ≤ 0.05.

## Discussion

To our knowledge this is the first study to document changes in maternal creatine metabolism during pregnancy. As creatine delivery to the placenta and fetus appears to be of maternal origin in precocial species, changes to maternal creatine homeostasis maybe a necessary adjustment of maternal metabolism to pregnancy in much the same way that alterations to glucose, amino acid, carbohydrate, lipid and fatty acid metabolism are required to support growing maternal tissues, the placenta and fetus [2].

### Changes to creatine demand during pregnancy development

Maternal plasma creatine concentrations and urinary creatine excretion were both significantly decreased in pregnancy. The rate of entry and removal of creatine into the plasma should be considered when interpreting these findings. Typically, entry of creatine into the maternal plasma would involve its absorption across the gut following ingestion of dietary creatine, and/or the release of creatine into the circulation from organs involved in *de novo* synthesis, mainly the liver [11]. Removal of circulating creatine may occur during pregnancy by the active transport of creatine into maternal tissues, the spontaneous (non-enzymatic) conversion of creatine to creatinine, excretion of creatine in the urine, or the transfer of creatine across the placenta [9,10,28,29].

The decreasing rate of urinary creatine excretion from mid to late gestation reported in the spiny mouse limits the probability that the decline in plasma creatine concentration observed in this study is the consequence of increased creatine loss through the kidney. However, the decreased renal excretion of creatine may be directly related to the observed fall in plasma (and hence, filtered) creatine. Whilst this study did not measure the activity of the creatine transporter in the kidney, no change in renal CrT1 gene expression was observed and therefore creatine uptake from glomerular filtrate may not be different between pregnant and non-pregnant spiny mice. Expression of monocarboxylate 12 (MCT12), a transport protein recently identified in kidney tubules and shown to perform facilitated diffusion of creatine was also not assessed in this study, but may play a role in the urinary creatine recovery observed with pregnancy in the spiny mouse [30].

The increased CrT1 gene expression identified in the heart and gastrocnemius muscle of the pregnant spiny mouse suggests these tissues have an increased capacity for creatine uptake from the circulation during pregnancy. Indeed, the pregnant heart had a significantly higher creatine content at term. These increases may reflect the need for increased phosphate buffering and preservation of ATP associated with the increased cardiac output of 40-50% during pregnancy [31,32]. The DEXA results indicate there was an increase in lean tissue mass of the pregnant spiny mouse by term. Previous studies in humans suggest that skeletal muscle contributes to 38-42% of lean tissue mass [33,34], and as skeletal muscle is the largest store of creatine in the body, this increased total mass together with the increase of CrT1 mRNA in pregnancy would be expected to result in increased creatine uptake from the maternal plasma into muscle beds. The adaptive value of the increased CrT1 expression may be to ensure that intracellular creatine levels remain at the levels required for normal muscle function. Mediators of increased CrT1 expression may be IGF-1 and triiodothyronine, both of which are up-regulated during pregnancy [35,36], and both of which have been shown to increase CrT1 expression and creatine uptake in skeletal myoblast cells by virtue of their effects on the Na^+^ transmembrane potential [37].

It is also likely that the placenta is an additional ‘sink’ for maternal plasma creatine, given that the creatine content of placental and fetal tissues increase across gestation [10,20]. What initiates changes in placenta CrT1 expression and creatine transfer during human pregnancy remains undetermined. The estrogen-related receptors (ERRα, β and γ) are highly expressed in the human placenta from the 2nd trimester and have been linked with energy metabolism within placental cells [38,39]. Recently, studies have described the role of ERRα in the up-regulation of CrT1 mRNA and creatine uptake in a L6 myotubule cell line [40]. Estrogen-related receptors may therefore be a prime candidate for facilitating creatine uptake into the placenta and maternal tissues during pregnancy. Interestingly, the spiny mouse dietary intake of creatine during pregnancy did not appear to change, suggesting that pregnancy may induce an increase in maternal endogenous creatine synthesis to meet the demand.

Maternal blood volume was not measured in this study, but would be expected to increase, as this is a fundamental, well-characterised adaptation that occurs in both rodent and human pregnancy [41,42]. Plasma expansion during pregnancy should thus be determined in the spiny mouse, to elucidate whether increased plasma volume might partially contribute to the decreased plasma creatine levels observed during pregnancy in this study. It is also unknown if creatine absorption across the gut is modified by pregnancy in the spiny mouse or the human. Changes to the gastrointestinal tract have been well characterised in rodent and human pregnancies, including the progesterone-induced decrease in gastrointestinal tract motility [43,44]. These adaptations facilitate increased nutrient absorption without an increase in dietary consumption [45]. As creatine absorption is limited to certain regions of the gut, intestinal transit time is thought to be a factor in creatine uptake under normal physiological conditions [46,47]. The slowed movement of substances through the intestine with pregnancy may therefore increase the contact time of creatine with CrT1 sites in the gut and increase intestinal absorption [48]. The uptake of creatine through the gut during pregnancy should thus be considered in future investigations of maternal creatine homeostasis.

### Adaptations to maternal creatine synthesis at term

Pregnancy in the spiny mouse was associated with a marked increase in maternal kidney AGAT mRNA and protein expression. As the rate limiting step of creatine synthesis, an increase in renal AGAT expression is considered a key indicator of increased endogenous capacity to synthesise creatine [28]. Whereas renal AGAT protein expression was greater in the kidney, hepatic GAMT protein expression (per μg of tissue protein) was decreased in pregnant spiny mice. This result suggests that the maximal rate of GAA methylation and thus creatine production in the liver may decrease with pregnancy, although this may be offset by the relatively large (29%) increase in liver size in the pregnant spiny mouse. Further work to establish maternal plasma levels of GAA during pregnancy, and of AGAT and GAMT enzyme activities *in vivo* of the maternal kidney and liver are needed to address these findings. Consideration of subsidiary sites of creatine synthesis such as the pancreas [49], and the changes to AGAT and GAMT expression observed in the brain, heart and gastrocnemius muscle identified in this study should also be considered when evaluating global maternal creatine production during pregnancy.

### Clinical translation

Establishing normative values (mean, range) for maternal plasma creatine and creatine urinary excretion during human pregnancy would be an important extension of these findings in the spiny mouse. Particular consideration should be given to the vegetarian population who obtain little creatine from their diet, and therefore require highly competent function of their renal-hepatic axis to synthesize endogenous creatine. Whether alterations to creatine metabolism have an association with obstetric complications such as poor fetal growth and placental insufficiency should also be considered in human-based studies. Recent literature suggests targeted dietary supplementation can play an essential role in reducing obstetric complications and neonatal mortality [50,51]. As a simple dietary intervention, creatine may have the ability to increase the cellular energy reserves of the mother, placenta and fetal organs, and may better outcomes following obstetric complications that lead to cellular energy failure and/or oxidative stress [52]. Indeed, previous studies conducted in the spiny mouse indicate increasing maternal dietary creatine improves neonatal outcomes following birth asphyxia [53-55].

## Conclusions

Pregnancy in the spiny mouse is associated with increased creatine content in the maternal heart and kidney, together with decreased plasma creatine concentration, decreasing urinary creatine excretion, and shifts in tissue-specific mRNA and protein expression of the enzymes AGAT and GAMT, and gene expression of CrT1. Whether maintenance of maternal creatine homeostasis is critical for fetal growth, and whether similar changes occur in human pregnancy is unknown and should be the focus of future studies.

## Abbreviations

AGAT: Arginine:glycine aminotransferase
ATP: Adenosine triphosphate
BMD: Bone mineral density
CrT1: Creatine transporter-1 protein
DEXA: Dual-energy X-ray absorptiometry
dGA: Days gestational age
ERR: Estrogen-Related Receptors
GAA: Guanidinoacetate
GAMT: Guanidinoacetate methyltransferase
IGF-1: Insulin-like growth factor 1

## References

[1] Butte NF, Wong WW, Treuth MS, Ellis KJ, Smith EOÄ (2004). Energy requirements during pregnancy based on total energy expenditure and energy deposition. Am J Clin Nutr.

[2] Herrera E (2000). Metabolic adaptations in pregnancy and their implications for the availability of substrates to the fetus. Eur J Clin Nutr.

[3] Lasuncion M, Lorenzo J, Palacin M, Herrera E (1987). Maternal factors modulating nutrient transfer to fetus. Neonatology.

[4] Bell AW, Bauman DE (1997). Adaptations of glucose metabolism during pregnancy and lactation. J Mammary Gland Biol Neoplasia.

[5] Herrera E, Knopp RH, Freinkel N (1969). Carbohydrate metabolism in pregnancy: VI. Plasma fuels, insulin, liver composition, gluconeogenesis, and nitrogen metabolism during late gestation in the fed and fasted rat. J Clin Investig.

[6] Zorzano A, Herrera E (1986). Comparative utilization of glycerol and alanine as liver gluconeogenic substrates in the fed late pregnant rat. Int J Biochem.

[7] Cetin I, Alvino G, Cardellicchio M (2009). Long chain fatty acids and dietary fats in fetal nutrition. J Physiol.

[8] Butte NF, King JC (2005). Energy requirements during pregnancy and lactation. Public Health Nutrition-Cab International.

[9] Ireland Z, Dickinson H, Snow R, Walker D (2008). Maternal creatine: does it reach the fetus and improve survival after an acute hypoxic episode in the spiny mouse (Acomys cahirinus)?. Am J Obstet Gynecol.

[10] Miller R, Davis B, Brent R, Koszalka T (2014). Transport of Creatine in the Human Placenta. Pharmacologist 1974.

[11] Wyss M, Kaddurah-Daouk R (2000). Creatine and creatinine metabolism. Physiol Rev.

[12] Brosnan JT, Brosnan ME (2010). Creatine metabolism and the urea cycle. Mol Genet Metab.

[13] Guimbal C, Kilimann M (1993). A Na (+)-dependent creatine transporter in rabbit brain, muscle, heart, and kidney. cDNA cloning and functional expression. J Biol Chem.

[14] Wallimann T, Wyss M, Brdiczka D, Nicolay K, Eppenberger H (1992). Intracellular compartmentation, structure and function of creatine kinase isoenzymes in tissues with high and fluctuating energy demands: the ‘phosphocreatine circuit’ for cellular energy homeostasis. Biochem J.

[15] Braissant O, Henry H, Villard A-M, Speer O, Wallimann T, Bachmann C (2005). Creatine synthesis and transport during rat embryogenesis: spatiotemporal expression of AGAT, GAMT and CT1. BMC Dev Biol.

[16] Dickinson H, Walker D, Cullen-McEwen L, Wintour E, Moritz K (2005). The spiny mouse (Acomys cahirinus) completes nephrogenesis before birth. Am J Physiol Renal Physiol.

[17] Brunjes P (1989). A comparative study of prenatal development in the olfactory bulb, neocortex and hippocampal region of the precocial mouse (Acomys cahirinus) and rat. Dev Brain Res.

[18] Lamers W, Mooren P, De Graaf A, Charles R (1985). Perinatal development of the liver in rat and spiny mouse. Its relation to altricial and precocial timing of birth. Eur J Biochem.

[19] Quinn TA, Ratnayake U, Dickinson H, Nguyen T-H, McIntosh M, Castillo-Melendez M (2013). Ontogeny of the adrenal gland in the spiny mouse, with particular reference to production of the steroids cortisol and dehydroepiandrosterone. Endocrinology.

[20] Ireland Z, Russell A, Wallimann T, Walker D, Snow R (2009). Developmental changes in the expression of creatine synthesizing enzymes and creatine transporter in a precocial rodent, the spiny mouse. BMC Dev Biol.

[21] Dickinson H, Walker D (2007). Managing a colony of spiny mice (Acomys cahirinus) for perinatal research. Australian and New Zealand Council for the Care of Animals in Research and Training (ANZCCART) News.

[22] Blake G, Knapp K, Fogelman I (2005). Dual X-ray Absorptiometry. Calcif Tissue Int.

[23] Watt K, Garnham AP, Snow RJ (2004). Skeletal muscle total creatine content and creatine transporter gene expression in vegetarians prior to and following creatine supplementation. Int J Sport Nutr Exerc Metab.

[24] Lowry O, Passonneau J, Lowry O, Passonneau J (1972). Typical fluorimetric procedures for metabolic assays. A Flexible System for Enzymatic Analysis.

[25] O’Connell BA, Moritz KM, Roberts CT, Walker DW, Dickinson H (2011). The placental response to excess maternal glucocorticoid exposure differs between the male and female conceptus in spiny mice. Biol Reprod.

[26] Speer O, Neukomm LJ, Murphy RM, Zanolla E, Schlattner U, Henry H (2004). Creatine transporters: a reappraisal. Mol Cell Biochem.

[27] Snow RJ, Murphy RM (2001). Creatine and the creatine transporter: A review. Mol Cell Biochem.

[28] Walker JB (1979). Creatine: biosynthesis, regulation, and function. Adv Enzymol Relat Areas Mol Biol.

[29] Forsum E, Forsberg A, Nilsson E, Bergström J, Hultman E (2000). Electrolytes, water, RNA, total creatine and calculated resting membrane potential in muscle tissue from pregnant women. Ann Nutr Metab.

[30] Abplanalp J, Laczko E, Philp NJ, Neidhardt J, Zuercher J, Braun P (2013). The cataract and glucosuria associated monocarboxylate transporter MCT12 is a new creatine transporter. Hum Mol Genet.

[31] Hunter S, Robson SC (1992). Adaptation of the maternal heart in pregnancy. Br Heart J.

[32] Thornburg KL, Jacobson S-L, Giraud GD, Morton MJ (2000). Hemodynamic changes in pregnancy. Semin Perinatol.

[33] Wang Z, Visser M, Ma R, Baumgartner R, Kotler D, Gallagher D (1996). Skeletal muscle mass: evaluation of neutron activation and dual-energy X-ray absorptiometry methods. J Appl Physiol.

[34] Heymsfield SB, Smith R, Aulet M, Bensen B, Lichtman S, Wang J (1990). Appendicular skeletal muscle mass: measurement by dual-photon absorptiometry. Am J Clin Nutr.

[35] Furlanetto R, Underwood L, WYK JV, Handwerger S (1978). Serum immunoreactive somatomedin-c is elevated late in pregnancy1. J Clin Endocrinol Metab.

[36] Osathanondh R, Tulchinsky D, Chopra IJ (1976). Total and free thyroxine and triiodothyronine in normal and complicated pregnancy. J Clin Endocrinol Metab.

[37] Odoom JE, Kemp GJ, Radda GK (1996). The regulation of total creatine content in a myoblast cell line. Mol Cell Biochem.

[38] Poidatz D, Dos Santos E, Brulé A, De Mazancourt P, Dieudonné M. Estrogen-related receptor gamma modulates energy metabolism target genes in human trophoblast. Placenta. 2012;33(9):688–95. 10.1016/j.placenta.2012.06.002 22763271

[39] Fujimoto J, Nakagawa Y, Toyoki H, Sakaguchi H, Sato E, Tamaya T (2005). Estrogen-related receptor expression in placenta throughout gestation. J Steroid Biochem Mol Biol.

[40] Brown EL, Snow RJ, Wright CR, Cho Y, Wallace MA, Kralli A, et al. PGC-1α and PGC-1β increase CrT expression and creatine uptake in myotubes via ERRα. Biochimica et Biophysica Acta (BBA)-Molecular Cell Research. 2014;1843(12):2937–43. 10.1016/j.bbamcr.2014.08.010 25173818

[41] Atherton J, Dark J, Garland H, Morgan M, Pidgeon J, Soni S (1982). Changes in water and electrolyte balance, plasma volume and composition during pregnancy in the rat. J Physiol.

[42] Pritchard JA (1965). Changes in the blood volume during pregnancy and delivery. Anesthesiology.

[43] Everson G (1992). Gastrointestinal motility in pregnancy. Gastroenterol Clin North Am.

[44] Wald A, Van Thiel DH, Hoechstetter L, Gavaler JS, Egler KM, Verm R (1982). Effect of pregnancy on gastrointestinal transit. Dig Dis Sci.

[45] Baron TH, Ramirez B, Richter JE (1993). Gastrointestinal motility disorders during pregnancy. Ann Intern Med.

[46] Peral M, Galvez M, Soria M, Ilundain A (2005). Developmental decrease in rat small intestinal creatine uptake. Mech Ageing Dev.

[47] Tosco M, Faelli A, Sironi C, Gastaldi G, Orsenigo M (2004). A creatine transporter is operative at the brush border level of the rat jejunal enterocyte. J Membr Biol.

[48] Salomons GS, Wyss M. (Eds.) Creatine and creatine kinase in health and disease. Springer Science and Business Media: Vol 46. 10.1007/978-1-4020-6486-9_16 18652084

[49] da Silva RP, Clow K, Brosnan JT, Brosnan ME. Synthesis of guanidinoacetate and creatine from amino acids by rat pancreas. The British journal of nutrition. 2014;111(4):571–7. 10.1017/S0007114513003012 24103317

[50] Lassi ZS, Salam RA, Haider BA, Bhutta ZA. Folic acid supplementation during pregnancy for maternal health and pregnancy outcomes. Cochrane Database Syst Rev. 2013, Issue 3:1 –71. 10.1002/14651858.CD006896.pub2 PMC10069458 23543547

[51] Neves AL, Guimarães AI, Rolão C (2012). Preventive oral iron supplementation for non-anemic women during pregnancy. Acta Obstet Ginecol Port.

[52] Dickinson H, Ellery S, Ireland Z, LaRosa D, Snow R, Walker DW (2014). Creatine supplementation during pregnancy: summary of experimental studies suggesting a treatment to improve fetal and neonatal morbidity and reduce mortality in high-risk human pregnancy. BMC Pregnancy Childbirth.

[53] Cannata DJ, Ireland Z, Dickinson H, Snow RJ, Russell AP, West JM (2010). Maternal creatine supplementation from mid-pregnancy protects the diaphragm of the newborn spiny mouse from intrapartum hypoxia-induced damage. Pediatr Res.

[54] Ireland Z, Castillo-Melendez M, Dickinson H, Snow R, Walker D (2011). A maternal diet supplemented with creatine from mid-pregnancy protects the newborn spiny mouse brain from birth hypoxia. Neuroscience.

[55] Ellery SJ, Ireland Z, Kett MM, Snow R, Walker DW, Dickinson H (2012). Creatine pretreatment prevents birth asphyxia-induced injury of the newborn spiny mouse kidney. Pediatr Res.

